# Evaluation of laparoscopic skills in medical students

**DOI:** 10.31744/einstein_journal/2022AO0091

**Published:** 2022-11-09

**Authors:** Fernanda Barma Leitzke, Marcelo Passos Teivelis, Leandro Luongo Matos, Nelson Wolosker, Daniel José Szor

**Affiliations:** 1 Faculdade Israelita de Ciências da Saúde Albert Einstein Hospital Israelita Albert Einstein São Paulo Brazil Faculdade Israelita de Ciências da Saúde Albert Einstein, Hospital Israelita Albert Einstein, São Paulo, SP, Brazil.; 2 Hospital Israelita Albert Einstein São Paulo Brazil Hospital Israelita Albert Einstein, São Paulo, SP, Brazil.

**Keywords:** Laparoscopy, Teaching, General surgery, Learning, Motor skills

## Abstract

**Objective:**

To evaluate the laparoscopic skills of medical students and identify personal characteristics in terms of greater easiness in performing laparoscopic surgical procedures.

**Methods:**

This study included medical students in the 6^th^ semester of a Medical School in Brazil who answered 10 questions concerning their habits and characteristics. A laparoscopic platform and an abdominal synthetic model were used to assess surgical skills comprising the three following surgical steps: to pass the needle through the trocar and to place it in the laparoscopic needle holder, to perform a laparoscopic simple stitch in synthetic liver parenchyma and, to perform a laparoscopic surgical knot. The duration of the activity was limited to four minutes and the procedure was monitored by a laparoscopic surgeon.

**Results:**

The study included 50 students. Of these, 18% completed the three surgical stages. Steps 1 and 2 were completed by 94% and 88% of students, respectively. No statistically significant variables were found when characteristics of the groups with and without success in the three stages were compared. The group that finished the activity had a faster time completing step 2 than the group that failed (mean time of 115.3 seconds against 157.8 seconds, p=0.03).

**Conclusion:**

The minority of students could complete effectively all three surgical steps. No personal traits related to greater surgical skill were identified.

## INTRODUCTION

Laparoscopic surgery is the standard procedure for various surgeries. This procedure was largely implemented in 1987 for cholecystectomy. Since this period, several advantages have been shown and currently they are reflected on surgical endpoints.^(1)^ Some of the benefits of the laparoscopic technique include less pain, minimum tissue aggressiveness and scarring, lower hospitalization, and faster recovery.^(2)^

The acquisition of surgical skills is a process that involves experience and repetition. Such skills should be reproduced in artificial models to ensure patient safety.^(3)^ The minimally invasive method has become the favored entry method for various procedures. Of note is that surgeons of newer generations seem to have better facility in assimilating certain methods such as laparoscopy.^(4)^ Habits such as playing video games, exercising, or playing musical instruments are associated with increased surgical skill.^(5)^

Most of the research concentrating on this topic evaluates physicians who have already graduated with some previous experience in surgery. The literature is scarce in assessing students or physicians with no experience. Therefore, assessing medical students who have never performed surgery and possess motor and sensory experiences exclusively from personal habits has the potential to demonstrate more accurately whether this association exists.

## OBJECTIVE

To evaluate the laparoscopic skills of students in the 6^th^ semester of Medicine and identify personal characteristics related to greater ease in performing laparoscopic surgical procedures.

## METHODS

The present study is a cross-sectional cohort formed by a convenience sample of students (n=50) from the 6^th^ semester of Medical School in Brazil. The authors sent all students an informed consent form and a virtual questionnaire. The questionnaire included 10 questions related to respondents’ personal and family characteristics and past and current personal habits.

The surgical skill assessment was performed in the medical school laboratory that included a video set with a monitor and 10mm and 30-degree surgical optics. The synthetic model of the abdominal cavity simulates intra-abdominal viscera. The liver was the organ chosen to pass the laparoscopic stitch to provide greater ergonomic comfort. Handedness was defined by writing hand preference.

The activity consisted of three surgical steps that are common to laparoscopy. These were to pass the needle through the trocar and place it in the laparoscopic needle holder, to perform a laparoscopic simple stitch in the synthetic liver parenchyma, and to perform a laparoscopic surgical knot.

A experienced surgeon in laparoscopy demonstrated the procedure in real-time before the student performed it. The time of the steps was measured in seconds with a maximum time of 4 minutes. After this period, the activity was closed regardless of the success of student in performing the procedure.

The laparoscopic material offered to the students contained a laparoscopic needle holder and a counter needle holder. The suture thread available was 2.0 needled cotton. No verbal or motor interference on the part of the surgeon was done during the undertaking of the activity by the student.

### Statistical analysis

The statistical analysis was based on the evaluation of subgroups, determined by the success or not in completing the three proposed steps. Therefore, two groups were formed. The measures of the categorical variables were expressed by absolute numbers and percentage, and for the continuous mean and standard deviation were used.

The normality of continuous variables was assessed using the Kolmogorov-Smirnov test. Comparison between categorical variables was performed using the Fisher’s test due to the small sample size and continuous variables using the Student’s *t*-test. A p value lower than 5% with two-tailed alternatives was considered significant.

### Ethics

This study was approved by the Research Ethics Committee of *Hospital Israelita Albert Einstein* (HIAE), CAAE: # 42188621.6.0000.0071; # 4.627.132. (SGPP: 4510-20). All participants signed the informed consent.

## RESULTS

The sample was composed mostly of female students (66%) with a mean age of 23.1 years. Most of part were dominant right hand (92%) and practice regular physical activity (76%). The habit of playing video games and musical instruments was present in 30% and 40% of the students, respectively. Previous participation as an observer or assistant in laparoscopic surgeries was 62% and 2%, respectively (Table 1).

**Table 1:** Characteristics of students that participated in the surgical skills evaluation

Variable		
Sex, n (%)			
	Male	17 (34)
	Female	33 (66)
Age (years)			
	Mean (Min-Max)	23.1 (20-40)
Handedness, n (%)			
	Right-handed	46 (92)
	Left-handed	3 (6)
	Both	1 (2)
Medical relatives, n (%)			
	Yes	23 (46)
	No	27 (54)
Medical parents, n (%)			
	Yes	12 (24)
	No	38 (76)
Exercise, n (%)			
	Yes	38 (76)
	No	12 (24)
Musical instruments, n (%)			
	Yes	20 (40)
	No	30 (60)
Electronic games, n (%)			
	Yes	15 (30)
	No	35 (70)
Previous participation in laparoscopic surgery, n (%)			
	Observer	31 (62)
	Participant	1 (2)
	No	18 (36)
Intended surgical career, n (%)			
	Yes	25 (50)
	No/not sure	25 (50)

Min-Max: Minimum-Maximum.

Fifty percent of the students intended to pursue a surgical career and 25% discarded this possibility. Of participants, 46% had a physician within their family, and for 24% of them at least one or both parents were physicians.

Eighteen percent of the students completed the three surgical steps within an average time of 187 seconds (ranging from 102 to 240 seconds). Duration of all steps were normally distributed. Steps 1 and 2 were completed by 94% and 88% of students, respectively (Table 2).

**Table 2:** Duration of surgical steps performed by medical students

Steps		Total time (in seconds)
Needle passage through trocar + correct positioning in needle holder		
	Step completed (% of students)	94
	Mean (Min-Max)	103.2 (30-200)
Passage of the needle through synthetic liver tissue		
	Step completed (% of students)	88
	Mean (Min-Max)	148.7 (39-240)
Knot confection		
	Step completed (% of students)	18
	Mean (Min-Max)	187.6 (102-240)

Min-Max: Minimum-Maximum.

When comparing the characteristics of the group with and without success in the three steps, no variable with statistical significance was found: sex (p=0.41), age (p=0.54), the practice of sports activity (p=0.09), musical instrument practice (p=0.2), the habit of playing video games (p=0.24), and the intention to pursue a surgical career (p=0.72) (Table 3).

**Table 3:** Students characteristics according to complete knot confection

Variables		No	Yes	p value
		n=41	n=9	
Sex*, n (%)				0.41
	Male	15 (30)	2 (4)	
	Female	26 (52)	7 (14)	
Age (years)#				0.54
	Mean±SD	22.8	21.7	
Exercise*, n (%)				0.09
	Yes	29 (58)	9 (18)	
	No	12 (24)	0	
Musical instruments*, n (%)				0.2
	Yes	19 (38)	1 (2)	
	No	22 (44)	8 (16)	
Video games*, n (%)				0.24
	Yes	15 (30)	1 (2)	
	No	26 (52)	8 (16)	
Intended surgical career*, n (%)				0.72
	Yes	20 (40)	5 (10)	
	No/not sure	21 (42)	4 (8)	
Step 1 (in seconds)#				0.22
	Mean±SD	107.8	85.7	
Step 2 (in seconds)#				0.03
	Mean±SD	157.8	115.3	

* Fisher exact test;

# Student´s *t*-test.

SD: standard deviation.

The group that completed the activity exhibited greater speed in the execution of step 2 when compared with the group without success (mean time 115.3 *versus* 157.8 seconds, p=0.03) (Table 3).

## DISCUSSION

No correlation was observed among previous or current behaviors, sex, age, intention to pursue surgical career in the future, and surgical performance regarding time to execute the procedures. The group that accomplished all steps required less time to pass the needle through synthetic liver tissue than those who could not complete the knot tying procedure (p=0.03).

The influence of video games on surgical skills is thoroughly discussed.^(6)^ An article published in 1992 in JAMA heralded the emerging of the “Nintendo surgeon” when laparoscopic cholecystectomy was gaining relevance.^(7)^ In that paper author claimed a subconscious competence that could be present in the mind of newer generations of surgeons.

Some skills developed while playing video games offer advantages in the surgical scenario. One is visual attention, including the ability to process information over time and the increased number of items that may be noticed and apprehended. In addition, spatial distribution throughout a visual field facilitates working in a 2D display while executing maneuvers that demand profundity.

A study demonstrated that surgeons who played games more than 3 hours/week made 37% fewer errors,^(6)^ performed 27% faster, and presented an overall scored 42% better than surgeons who never played video games before. Authors also assessed the impact of video games just before surgical practice. They reported that surgeons who performed a warm-up playing Playstation 2 video game for 18 minutes before a laparoscopic activity conducted laparoscopic skills faster than the Control Group. They also concluded that the warm-up exercise would enhance performance and suppress technical errors.

Our data showed no correlation between playing video games and surgical performance (p=0.24). As argued elsewhere, the mechanics of video games, including game design, platform, and utilization of remote control, may have an effect as crucial as the amount of time and/or type of content played. We noticed that students played different video games, including those presented in mobile phones. Maybe these game platforms do not affect hands and visual performance like those played on the television or computers. Although, it is worth citing a research by Awal et al., that offered a laparoscopic smartphone simulator to medical students, and those students who trained. The students who trained in this simulator to medical students, and those students who trained presented better laparoscopic skills in the laparoscopic box compared with those who did not play using smartphones.^(8)^

Our data did not reveal improved performance among students who played video games, musical instruments, and/or exercised. Nevertheless, other studies proved the benefits of skill transferring from these activities to surgical performance. Perhaps these studies demonstrated a correlation, one cannot establish a definitive causality.

There is a tendency in published literature to report a lower surgical skill level of left-handed medical students.^(9)^ The predominance of right-handed surgeons and thus right-handed instruments is a challenge to those students who are left-handed. In the present research, left-handed students showed higher levels of ambidexterity, including some activities practiced exclusively with right hand.

In our data, participant’s interest in surgery was not correlated with better surgical skills, although almost 50% of the students intend to be surgeons. There are different results reported in the literature concerning this topic. Cheng Luo et al. reported that medical students who reported interest in a future surgical career in the last years before graduation had a better performance in laparoscopic tasks.^(10)^ Contrary, Lee et al., did not find better dexterity among medical students who were interested in pursuing a surgical career.^(11)^ Students included in our study were from the middle of graduation on. Consequently, at this point, although they reported interest in surgery, fewer opportunities to observe and participate in surgical procedures were made available. Perhaps, this justify the reason that their ability did not enhance despite their interest.

Students who accomplished all the steps took less time to perform the first two steps. These students had a higher ability with laparoscopic movements compared with others who could not complete the activity. Since they achieved a knot tying procedure, it is reasonable that less complex steps, such as needle manipulation and passing the point through the stomach, were done faster.

This study presents some limitations. First, the number of students was modest and results may not reflect the conclusions if we analyze the totality of students. Second, the evaluation itself had certain biases. Since groups of students did the activity, one could witness the others performing the procedures, and they might absorb the most prevalent errors, therefore, avoiding such errors when they execute the procedure. Also, the time of the activity was limited to at most 4 minutes for each student due to the short time available for using the laboratory.

Time limitation may also play an important role in the finding that why our results were discordant with most articles published in the literature. We could not find a characteristic related to better dexterity. Time pressure may affect the performance and reduce the gap between students regarding individual skills. Some students reported high anxiety levels once they were being evaluated, which probably influenced the outcomes. Spaced time evaluations are reported as responsislbe tto reduce procedure-related anxiety and improve outcomes.^(12)^

## CONCLUSION

The minority of students could complete effectively all three surgical steps. No personal characteristics responsible to improve surgical dexterity were identified.
